# Supplementary figures and images for: Comprehensive analysis of different tumor cell-line produced soluble mediators on the differentiation and functional properties of monocyte-derived dendritic cells
Source: PLoS One. 2022 Oct 4;17(10):e0274056. doi: 10.1371/journal.pone.0274056 (PMC9531813; doi:10.1371/journal.pone.0274056)

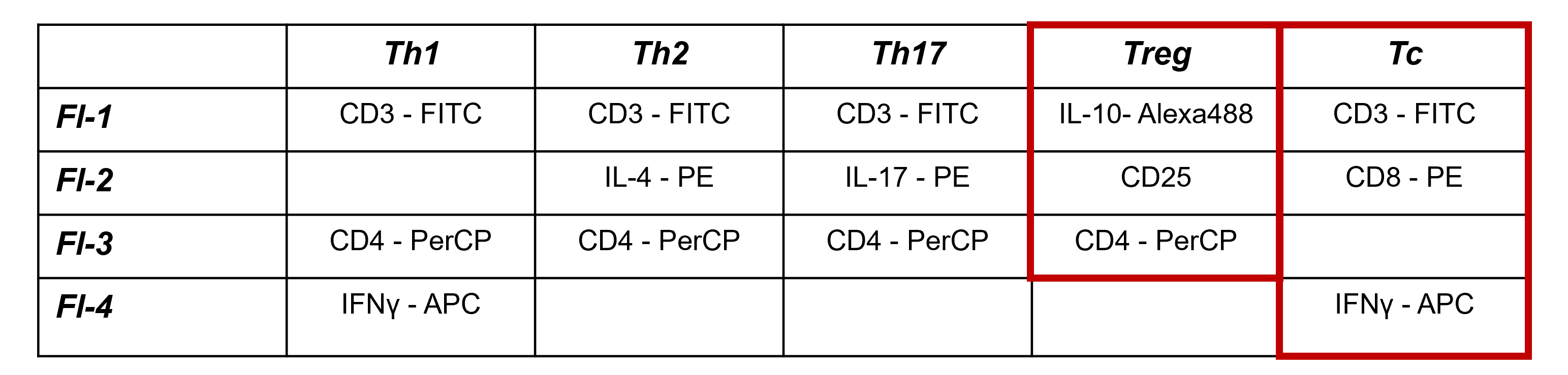

Supplement: S1 Fig — The vesicular transport was inhibited by BD GolgiStop™ protein transport inhibitor (BD Biosciences) after the activation. The cells were then labeled with anti-human CD3-FITC, and anti-human CD8-PE or CD4-PerCP and anti-human CD25-PE antibodies. The samples were then fixed and permeabilized and labeled with ananti-human CD8-PE or CD4-PerCP and with anti-human IFNγ-APC, anti-human IL-4-PE, anti-human IL-10-Alexa Fluor 488, anti-human IL-17-Pe antibodies. Fluorescence intensities were measured by Novocyte2000R Flow Cytometer. (TIF) [file pone.0274056.s001.tif]

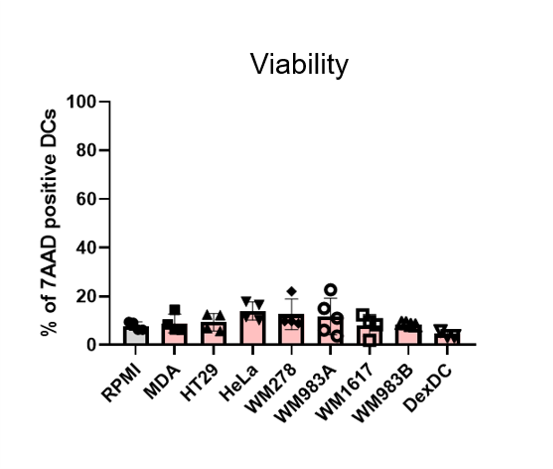

Supplement: S2 Fig — Cell viability was assessed by 7-aminoactinomycin-D (7-AAD; 10 μg/ml) staining. Samples were stained for 15 minutes with 7-AAD immediately before flow cytometric analysis. Fluorescence intensities were measured by Novocyte2000R Flow Cytometer, and data were analyzed by the FlowJo v X.0.7 software. (TIF) [file pone.0274056.s002.tif]

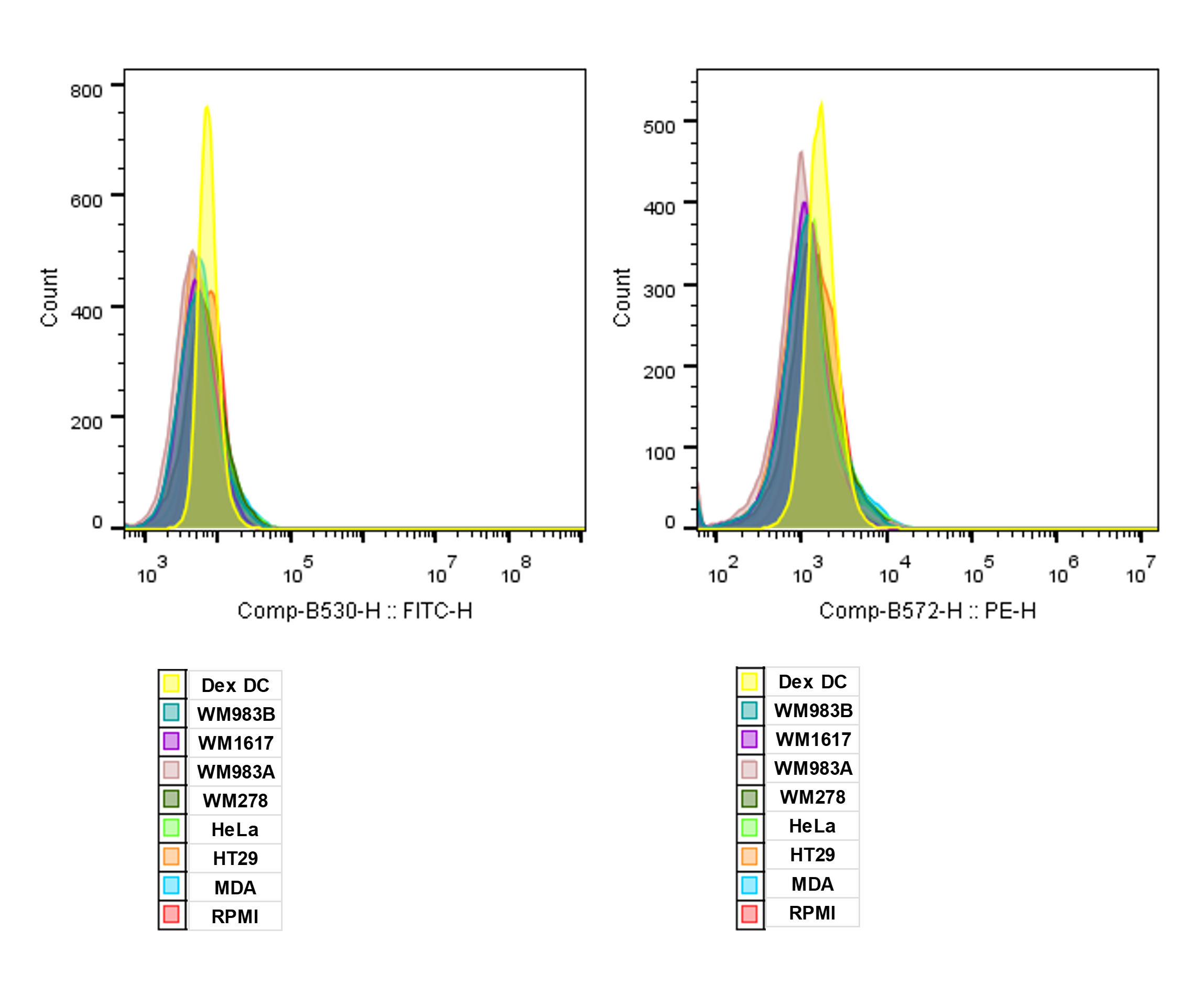

Supplement: S3 Fig — CD14+ monocytes were cultured with 100 ng/ml IL-4, 80 ng/ml GM-CSF ± MDA-MB231-, HeLa-, HT29-, WM278-, WM1617-, WM983A-, WM983B-CM or in the presence of dexamethasone for four days. On day 4, the autofluorescence of moDCs was measured by flow cytometry. Histograms show one representative experiment. (TIF) [file pone.0274056.s003.tif]

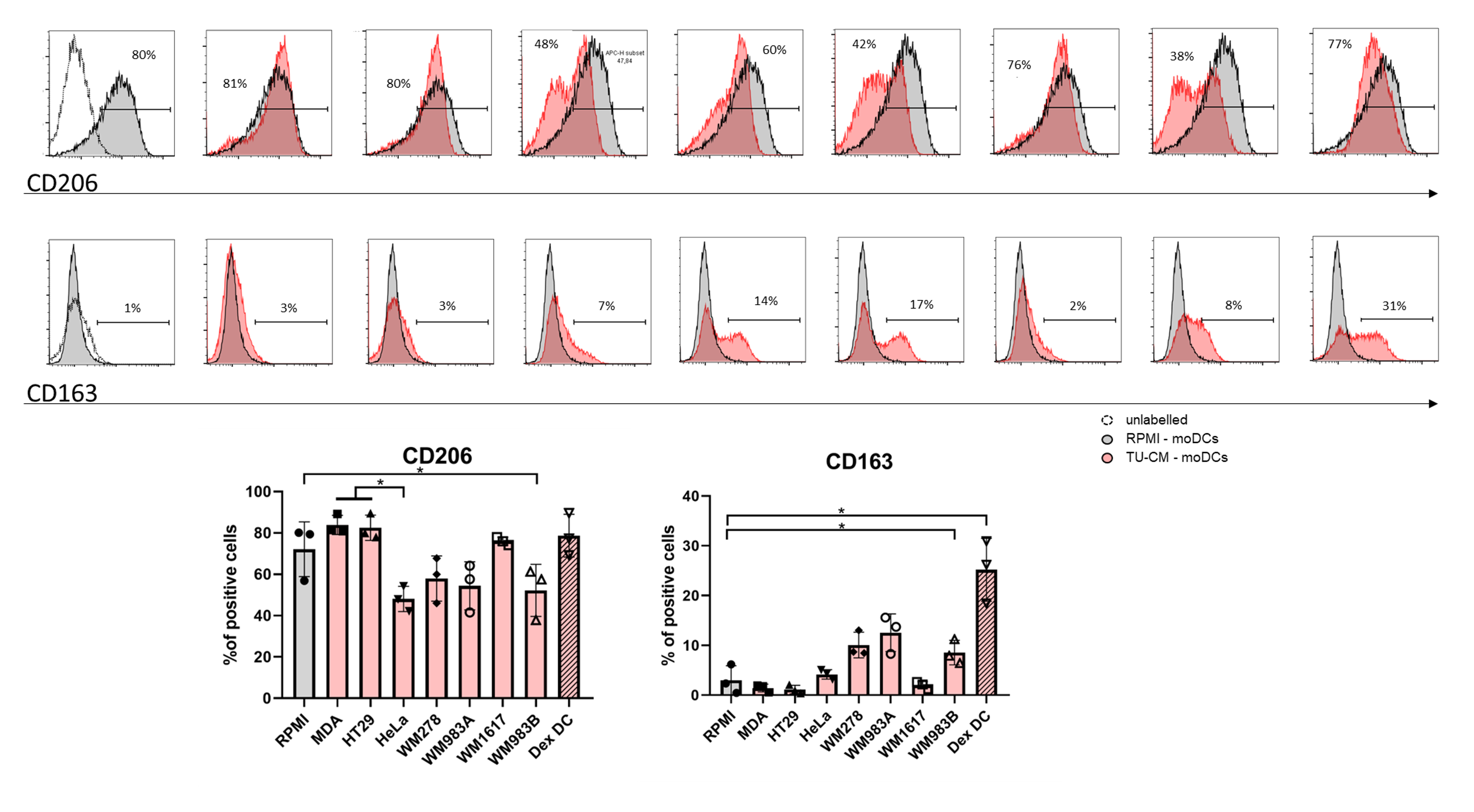

Supplement: S4 Fig — CD14+ monocytes were cultured with 100 ng/ml IL-4, 80 ng/ml GM-CSF ± MDA-MB231-, HeLa-, HT29-, WM278-, WM1617-, WM983A- or WM983B-CM for four days. On day 4, the expression of CD163 and CD206 were analyzed by flow cytometry. Figures show the mean plus SD values of the populations positive for the measured surface molecules calculated from three experiments. Histograms show one of the three independent experiments. Significance is indicated by *P < 0.05. (TIF) [file pone.0274056.s004.tif]

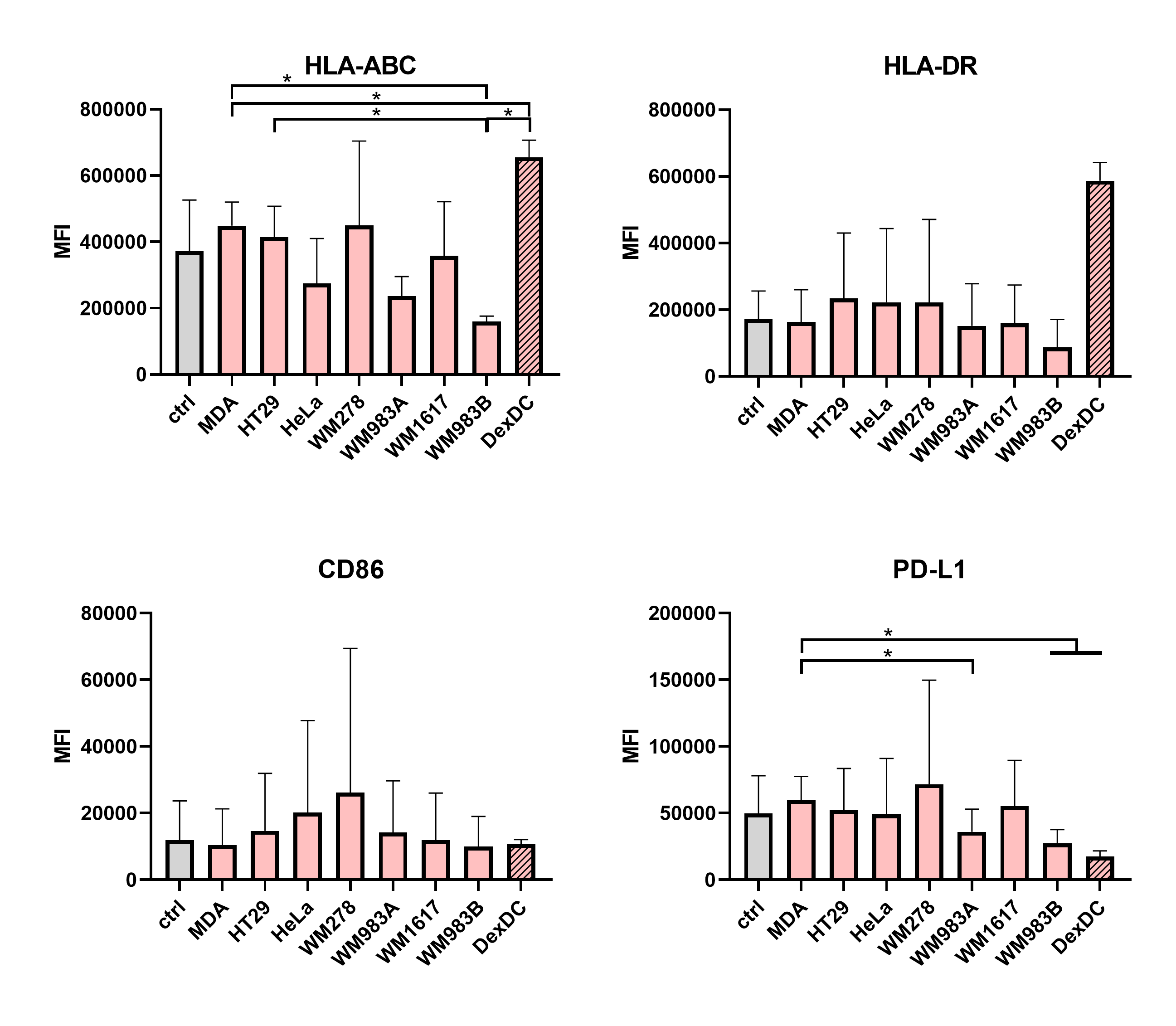

Supplement: S5 Fig — CD14+ monocytes were cultured with 100 ng/ml IL-4, 80 ng/ml GM-CSF ± MDA-MB231-, HeLa-, HT29-, WM278-, WM1617-, WM983A- or WM983B-CM for four days. On day 4, the cell surface expression of HLA-ABC, HLA-DR, CD86, and PD-L1 was analyzed by flow cytometry. The figure shows the MFI plus SD calculated from five independent experiments. Significance is indicated by *P < 0.05. (TIF) [file pone.0274056.s005.tif]

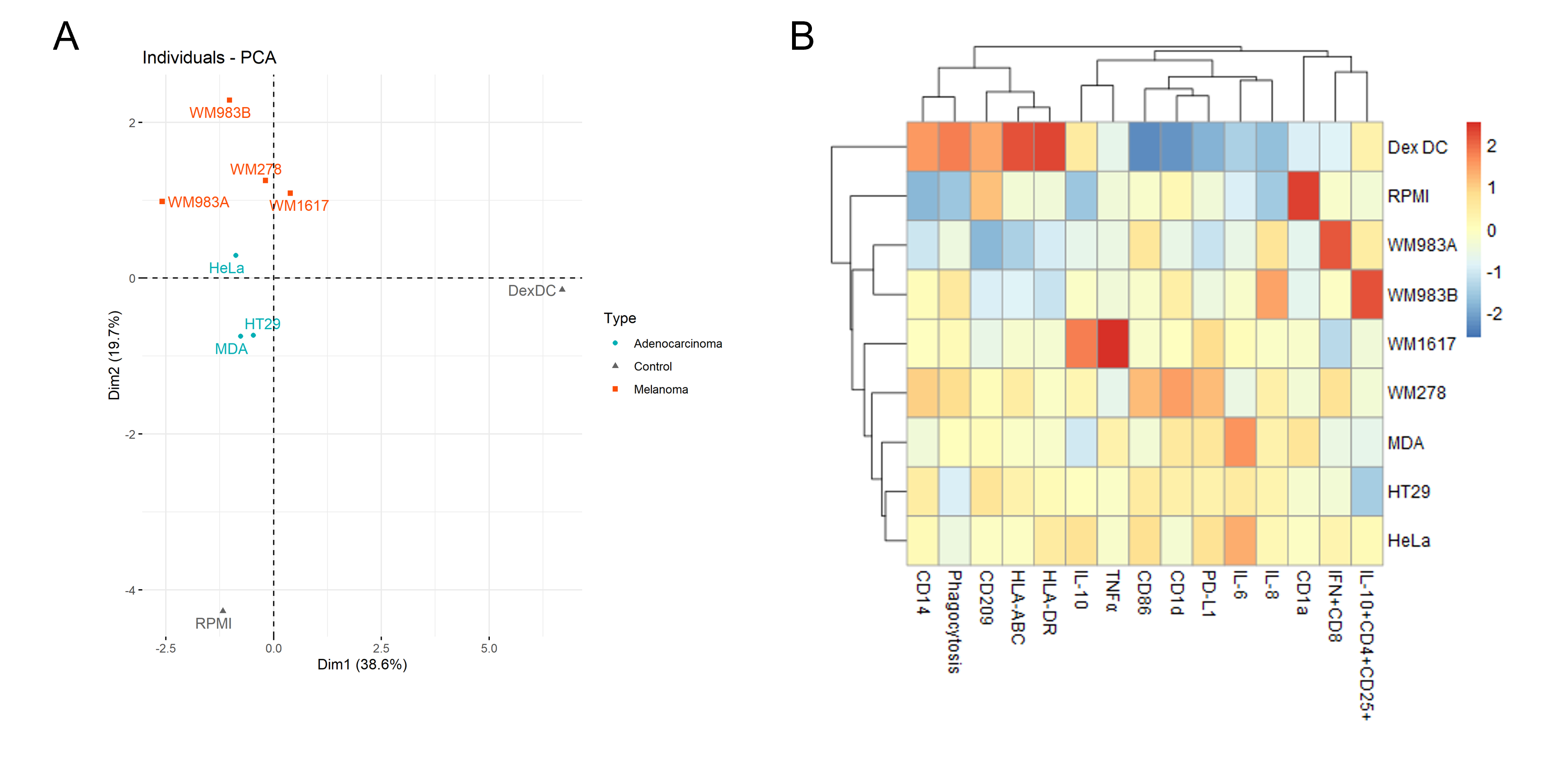

Supplement: S6 Fig — Color-coded projection of the dataset obtained from adenocarcinomas- (blue circles) and melanomas- (red squares) conditioned and control (grey triangles) moDCs on a two-dimensional PCA (A). Z-score values were calculated from the raw data, and a column-scaled heatmap (B) was generated. (TIF) [file pone.0274056.s006.tif]

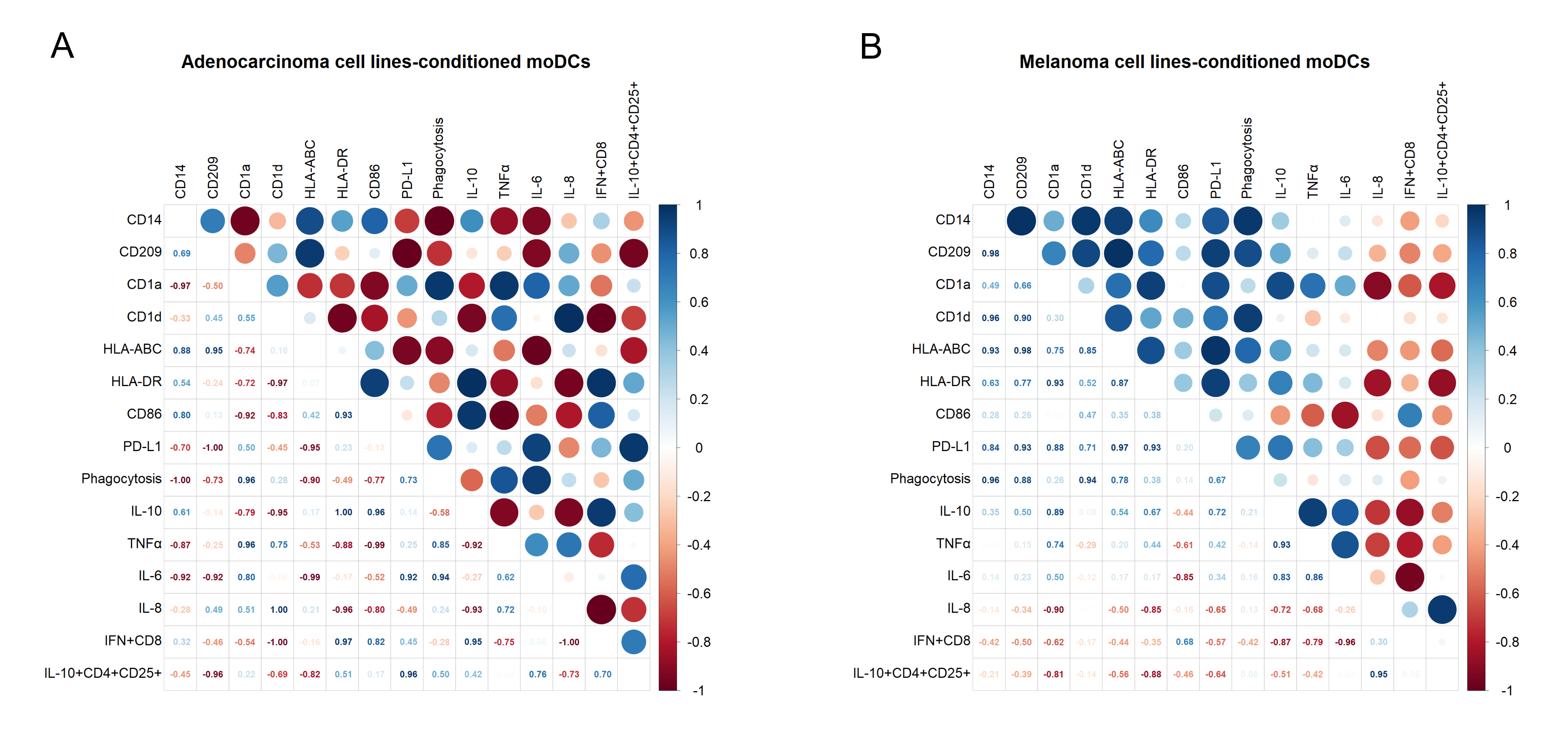

Supplement: S7 Fig — Panel A demonstrates a mixed correlogram from the data of the three adenocarcinoma-educated moDCs without controls. On the lower part, the individual correlation matrix values are shown. Blue circles indicate positive correlation on the upper part, while negative correlations are displayed in red. The color intensity and the size of the circle are proportional to the correlation coefficients (Pearson’s correlation). The correlation between the four melanoma cell line-educated DCs is shown in Panel B. On the lower part, the individual correlation matrix values are shown. Blue circles indicate positive correlation on the upper part, while negative correlations are displayed in red. The color intensity and the size of the circle are proportional to the correlation coefficients (Pearson’s correlation). (TIF) [file pone.0274056.s007.tif]
