# Supplementary material for: Comprehensive analysis of different tumor cell-line produced soluble mediators on the differentiation and functional properties of monocyte-derived dendritic cells
Source: PLoS One. 2022 Oct 4;17(10):e0274056. doi: 10.1371/journal.pone.0274056 (PMC9531813; doi:10.1371/journal.pone.0274056)
Supplement: Attachment — Submitted filename: Response to Reviewers.docx [file pone.0274056.s008.docx]

**Response to Reviewers**

Thank you very much for the review of our manuscript entitled *“Comprehensive analysis of different tumor cell-line produced soluble mediators on the differentiation and functional properties of monocyte-derived dendritic cells ”* and the opportunity to resubmit a revised version.

We greatly appreciate the positive evaluation of our manuscript and the valuable comments of the Reviewers. We addressed the comments of the reviewer. To enhance the quality of our manuscript we have conducted additional experiments and made several changes in the text. Please see our detailed responses below.

**Reviewer#1**:

**Major revisions:**

**1)** There are several inconsistencies between the text and the results presented in the figures. In particular:

First of all, we are very grateful to the reviewer for a thorough review of our manuscript.

We apologize for the inconsistencies between the text and the results presented in the figures. We corrected the highlighted sentences, refined the statements and conclusions. We checked the consistency of the text and figures as well as the text were edited in the revised manuscript.

“HT29-, HeLa-CM-moDCs and in accordance with the literature, DexDCs also showed significantly higher CD14 expression [23] compared to the control moDCs (Fig 1A).” (lines 270-271): Fig 1 does not show significant result for HeLa cell line.

We corrected the text (lines 287-290): *“HT29-, WM278-CM-moDCs and in accordance with the literature, DexDCs also showed significantly higher CD14 expression [26] compared to the control moDCs (Fig 1A)”.*

“HT29, WM278, WM1617, and WM983B-conditioned moDCs expressed significantly more CD14 and CD209 molecules than their MDA- or WM983A-educated counterparts.” (lines 280-282): This should be re-worded to refer to higher frequency of co-expression of CD14 and CD209 instead of more expression. Fig 1 also does not show that the difference between WM1617 and MDA or WM983A cell lines is significant.

We corrected the following sentences (lines 297-299): *“HT29, WM278 and WM983B-conditioned moDCs showed higher frequency of co-expression of CD14 and CD209 molecules.”*

“… significant difference was observed between the WM983A and DexDC groups).” (line 293): this difference is between DexDC and WM278 and not between DexDC and WM983A.

Corrected: (lines 308-310)

*“In general, the expression of CD1d on moDCs was not significantly modulated by tumor cells; while a significant difference was observed between the WM278 and DexDC groups.”*

“… the phagocytic capacity was increased slightly but not significantly compared to control moDCs. These results indicate that only certain tumors can alter the phagocytic capacity of the DCs.” (line 374-376) and “… indicating that tumor microenvironment influences the phagocytic capacity of DCs” (line 522): The authors state that the results indicate that only certain tumors may impair or influence the phagocytic capacity of DCs, stating that no significant results are shown. I think the authors should be less categorical in the Discussion.

We corrected this sentence taken into account the suggestion of the Reviewer and the data gained from the new, additional results.

In the Results the statement was corrected: (lines 401-405)

*“A significant difference can be detected in the phagocytic activity of the control moDCs and WM278, WM1617 or DexDCs, as well as between the HT29- and WM278-CM-conditioned monocyte-derived cells. These results may indicate that certain tumors can alter the phagocytic capacity of the DCs**.”*

In the Discussion the statement was corrected: (lines 581-584)

*“We revealed differences in the uptake of Lactobacillus casei by tumor cell line-conditioned moDCs, which shows that how the tumor microenvironment in some cases could influences the phagocytic capacity of DCs.”*

Fig 5 needs more thorough description of gating. Panel A would be better visualized as a 2D plot for CD4 and CD8 instead of independent density/histogram plots. For the quantification on the right it is not entirely clear what the denominators are. CD8+ IFNg+ should probably be reported as a percentage of total CD8+ cells and similarly CD4+CD25+FOXP3+ should be percentage of total CD4+. It is not clear whether CD4+CD25+IL10+ should be reported as percentage of CD4+ or CD4+CD25+.

We apologize for the incorrect methodological description and evaluation. In the revised version of our manuscript, we present the effect of conditioned moDCs on the T cell response more precisely and we optimized the gating strategy **(Figure 5)**. Additionally, according to the Reviewer’s recommendation, we re-analyzed our flow cytometry results. Consequently, our row data for the statistical and bioinformatic analysis were also changed, thus we performed the new bioinformatic analysis taking into account the new data. New bioinformatics analysis is presented in Figure 6, S6 and 7 Figs. As the cells were not co-labeled with anti-human CD8 and CD4 we can not provide 2D plot. For a better understanding, a new supplementary figure **(S1 Fig)** was created about the staining panel:

As the staining with PerCP and APC parallel generates compensation problems, and due to other technical difficulties we cannot improve the FoxP3 staining. Therefore, these data were excluded in the revised version of the manuscript.

As in the case of Th1, Th2 and Th17 cells we did not identify changes, our figure depicts the IL-10 producing CD4^+^CD25^+^ cells as well as the IFNγ- producing CD4^+^ T cell response (staining panels in red frames in the table). In **Figure 5** of the revised version, the bar graphs show the frequency of IFNγ positive cells in the CD8^+^ T cell population and the bar graphs show the frequency of IL-10 positive cells in the CD4^+^CD25^+^ cell population.

Related, corrected results: (lines 431-442)

*“Similarly, we have detected inhibited cytotoxic T cell activation in the presence of WM983B metastatic melanoma cell line-educated moDCs; in contrast, its primary counterpart induced the activation of IFNγ-producing CD8+ T cells (Fig 5). Additionally, we measured the intracellular level of IL-10 in the CD4+CD25+ subpopulation (Fig 5). Interestingly, in opposted to the WM1617metastatic melanoma cell line, WM983B-educated moDCs were prone to enhance the IL-10 production of CD4+CD25+ PBL. We detected significant differences in the IL-10 producing PBL activation capacity between metastatic melanoma cell lines and MDA/HT29 adenocarcinoma cell lines. Additionally, HT29 had the significantly lower capacity to induce IL-10 production by CD4+CD25+ PBL (Fig 5) than the HeLa adenocarcinoma cell line. According to our data, the T-cell polarization ability of DCs altered by different tumor cell lines promotes the development of IFNγ or IL-10 producing T cells, but different tumors induce diverse responses.”*

“Based on our correlation analysis, between HLA-ABC, HLA-DR and PD-L1, their expression was similarly regulated in the case of melanoma cell lines-educated DCs, whereas strong negative correlation was observed in the group of moDCs conditioned by adenocarcinomas with the respect of HLA-ABC, HLA-DR and PD-L1 expressions.” (lines 539-543): Regarding adenocarcinoma cell lines, only HLA-ABC and PD-L1 are strongly anti-correlated, the other pairs (HLA-ABC with HLA-DR and HLA-DR with PD-L1) are just not correlated. This should be described more accurately.

New data were generated for the bioinformatic analysis as we increased the donor number, re-evaluated the T cell response-related results and control data (RPMI and DexDC) were excluded. Bioinformatic analysis was performed again as a consequents of these changes. The complete chapter “*Principal component analysis of the phenotypical and functional properties of moDCs differentiated in tumor cell lines-derived conditioned media*” was carefully re-written. (Lines 456-519)

*“To examine the overall effect of dexamethasone or the cancer cell lines-derived supernatants on the moDCs, principal component (PCA) and correllation analysis were performed by using the data from flow cytometry and ELISA (Fig 6, S6 S7 Figs). The analysis revealed that tumor cells-educated moDCs were well distinguished from dexamethasone-treated or control (RPMI) DCs (S6 Figs A and B). To reveale differences in the strategy of melanomas and adenocarcinoma cell lines in the manipulation of moDC differentiation Therefore, the control moDCs (RPMI and DexDC) were not analyzed in the correlation analysis using heatmaps and correlograms (Fig 6, S6, S7 Figs). The bioinformatic analysis revealed, that in generall, melanomas and adenocarcinomas generally induce different changes in monocytes derived cells. However WM1617 metastatic melanoma cell line has the same effect on the moDC’s function as the adenocarcinomas, but it is quite differ from the other melanomas (Fig6 panels A and B). Two groups were created; group of moDCs differentiated in the presence of adenocarcinomas (Fig 6C, S7 Fig A) or melanomas (Fig 6D S7 Fig B). The correlation analysis revealed significant differences in the features of adenocarcinomas- or melanomas-edited moDCs. Stronger positive or negative correlations can be identified between the phenotypical or functional properties in the case of the moDCs differentiated in adenocarcinoma- than in the group of melanomas-conditioned moDCs. Based on our analysis, positive correllations were observed in the group of adenocarcinoma-educated monocyte-derived cells: between 1) CD14 and CD209, HLA-ABC, HLA-DR, CD86, and IL-10 production, between 2) CD209 and HLA-ABC, between 3) CD1a and CD1d, HLA-ABC, PD-L1, phagocytic activity, and TNFα/ IL-6/ IL-8-production, between 4) CD1d and TNFα/ IL-8-production, between 5) HLA-DR and CD86, IL-10 production and IFNγ-producing CD8+ T cells activatory potential, between 6) CD86 and IL-10 production and IFNγ-producing CD8+ T cells activatory capacity, between 7) PD-L1 and phagocytic activity, IL-6 secretion and IL-10-producing CD4+CD25+ cells activatory capacity, between 8) phagocytosis and TNFα/ IL-6-production, between 9) IL-10 release and IFNγ-producing CD8+ T cells activatory capacity, between 10) TNFα production and IL-6/IL-8 secretion, between 11) IL-6 and IL-10-producing CD4+CD25+ cells activatory capacity, and finally and surprisingly, between 12) the IFNγ-producing CD8+ T cells and IL-10-producing CD4+CD25+ cells activatory capacity (Fig6C and S7 Fig panel A). Secondly, negative correllations were observed in the group of adenocarcinoma-educated monocyte-derived cells: between 1) CD14 and CD1a, PD-L1, phagocytic activity and TNFα/ IL-6-production, between 2) the CD209 and CD1a, PD-L1, phagocytic activity, IL-6-production and IL-10-producing CD4+CD25+ cells activatory capacity, between 3) the CD1a and HLA-ABC, HLA-DR, CD86, IL-10 secretion and IL-10-producing CD4+CD25+ cells activatory capacity, between 4) the CD1d and HLA-DR, CD86, IL-10 secretion IFNγ-producing CD8+ T cells and IL-10-producing CD4+CD25+ cells activatory capacity, between 5) the HLA-ABC and PD-L1, phagocytic activity, TNFα/ IL-6-production and IL-10-producing CD4+CD25+ cells activatory capacity, between 6) the HLA-DR and phagocytic activity, TNFα-production and IFNγ-producing CD8+ T cells activatory capacity, between 7) the CD86 and phagocytic activity and TNFα/IL-6/IL-8-production, between 8) the phagocytosis and IL-10 production, between 9) the IL-10 secretion and TNFα/IL-8-production, between 10) the TNFα and IFNγ-producing CD8+ T cells activatory capacity, finally, between the IL-8 production and T cell activator capacity (Fig6C and S7 Fig panel A).*

*We continue with the description of melanoma cell lines-conditioned moDCs (Fig6D and S7 Fig panel B). Firstly, positive correllations were observed in the group of melanoma-educated monocyte-derived cells: between 1) CD14 and CD209, CD1a, CD1d, HLA-ABC, HLA-DR, PD-L1 and phagocytosis, between 2) CD209, CD1a, CD1d, HLA-ABC, HLA-DR, PD-L1, phagocytic activity and IL-10 production, between 3) CD1a and HLA-ABC, HLA-DR, PD-L1 and IL-10/TNFα/IL-6 production, between 3) CD1d and HLA-ABC, HLA-DR, CD86, PD-L1 and phagocytic activity, between 4) HLA-ABC and HLA-DR, PD-L1, phagocytic activity and IL-10 production, between 5) HLA-DR and PD-L1, phagocytic activity and IL-10/ TNFα production, between 6) CD86 and IFNγ-producing CD8+ T cells activatory capacity, between 7) PD-L1 and phagocytic activity and IL-10/ TNFα production, between 8) IL-10 and TNFα/IL-6 production, between 9) TNFα secretion and IL-6, finally, between 10) IL-8 production and T cell activation (Fig6D and S7 Fig panel B). Secondly, negative correllations were observed in the group of melanoma-educated monocyte-derived cells: between 1) CD14 and IL-10-producing CD4+CD25+ cells activatory capacity, between 2) CD209, CD1a, HLA-ABC, HLA-DR, PD-L1, IL-10 or TNFα and IL-8 production and T cell stimulatory potential, between 3) CD86 and TNFα/IL-6 production, and finally, between 4) IL-6 and IL-8 production and IFNγ-producing CD8+ T cells activatory capacity (Fig6D and S7 Fig panel B). The detailed analysis of the different correlations observed between each marker in the two groups is listed in S7 Fig. In summary, PCA analysis revealed differences in the strategy of melanomas and adenocarcinoma cell lines in the manipulation of moDC differentiation, thus in their ability to orchestrate T cell responses.”*

**2)** There are some errors in the annotation of the figures and the accompanying descriptions. I encourage the authors to review the annotation of their figures and their descriptions. In particular:

Lines 291 and 301 refer to Fig 1C, however, Figure 1 contains only two parts (Fig 1A and Fig 1B).

We apologize for this mistake, it was corrected in the new version of the manuscript. In parallel to correcting this error, all references and annotations to the figures were checked.

Presenting Fig 3B as a heatmap makes it very difficult to compare with Fig 3A. Can another visualization be found (e.g. perhaps the same as Fig 3A) that makes these results easier to interpret?

Thanks you for your constructive suggestion! To make easier the comparison of the results of Figure 3A with Figure 3B, we prepared a column diagram also from Figure 3B (**Figure 3B**) which demonstrates the production of a soluble mediator of tumor cell lines prior to their use for the monocyte precondition.

The references to Fig 5 do not target the correct part of the figure. For example, the reference to Fig 5C on line 408 should probably be in the previous sentence and replaced here by Fig 5D.

Thank you for bringing these errors to our attention. In the revised version of the manuscript, the text was aligned with the figures and panels in all cases.

There is an annotation problem between Fig 6 and its description (the description mentions Correlogram B and C while the correlograms make up Fig 6C and Fig6D).

Thank you for bringing these errors to our attention. In the revised version of the manuscript, the text was aligned with the figures and panels in all cases.

**3)** The authors propose an interesting approach using bioinformatics to enrich the interpretation of their results. Although the authors have the good idea to use the correlation circle as a complement to the PCA, they did not apply it on the variables (as described in the figure description) but on the cell lines. Correcting this error and interpreting the results obtained could bring possibly interesting information and help to enrich the bioinformatic results.

We are grateful to the Reviewer for drawing this error to our attention. In the corrected version of the manuscript, the variables are applied to visualize the correlation analysis.

The corrected figures: **Figure 6 panels C and D, S7 Fig panels A and B**.

Bioinformatic analysis was performed again as a consequents of new data, as well as the complete chapter of ‘Results’ focusing to the bioinformatics was also re-written (Lines 458-521).

“In summary, PCA analysis revealed differences in the strategy of melanomas and adenocarcinoma cell lines in the manipulation of moDC differentiation, thus in their ability to orchestrate T cell responses.” (lines 449-451): PCA shows clear differences between the cell lines and the control (RPMI and DexDC) but it doesn’t show differences between the different cell lines. Can the authors perhaps provide a PCA after removing the RMPI and DexDC controls to see if it validates this conclusion?

We are extremely grateful to the Reviewer for this comment. We performed further bioinformatic analysis of our data which revealed differences between the different tumor cell-lines-conditioned moDCs. To show these results, we provide a PCA, heatmap, and correlation analysis after removing the RMPI and DexDC controls **(Figure 6 panels A, B, C and D, S7 Fig panels A and B)**. These results now clearly support/ validate this conclusion:

“In summary, PCA analysis revealed differences in the strategy of melanomas and adenocarcinoma cell lines in the manipulation of moDC differentiation, thus in their ability to orchestrate T cell responses.”

A complete analysis including data from control (RPMI, DexDC) samples is also presented as an supplementary figure **(S6 Fig panel A and B)**.

We re-analyzed the results, as well as the Results session was also re-written (Lines 454-517).

**Minor revisions:**

**1)** A better description of the method of the bioinformatics part would be helpful (package version, function parameters, ...).

We agree with the reviewer and we provided a more detailed description of the method of the bioinformatics part in the material and methods: (lines 254-267)

*“Bioinformatical Correlation analyses R (version 4.1.3) [15] and RStudio (version 1.4.1717) were used for bioinformatical analyses. The dataset contained the mean of marker expression, cytokine secretion, T-cell polarizing, and phagocytosis data. Correlation matrices were performed by R base package (v 4.1.3) with cor function for adenocarcinoma and melanoma cell lines-conditioned moDCs separately. Correlograms from correlation matrices were plotted by corrplot (v 0.92) package [16] with corrplot function. P-value matrix and confidence intervals matrix were added using the cor.mtest function of corrplot package. Significance levels were defined as *P < 0.05; **P < 0.01; ***P < 0.001. Mixed correlograms were performed by corrplot.mixed function of the corrplot package. Heatmap was generated from the raw data by pheatmap function of pheatmap (v 1.0.12) library [17]. Principal Component Analysis (PCA) was made by PCA function of FactoMiner package (v 2.4) [18] from raw data with or without controls (RPMI and dexDC) separately. Results of the PCA were visualized by factoextra package (v 1.0.7) [19] with fviz_pca_ind function.”*

New references:

*“R Core Team. R: A Language and Environment for Statistical Computing [Internet]. Vienna, Austria; 2021. Available from:* [*https://www.r-project.org/*](https://www.r-project.org/)*” (16)*

*Kolde R. Package “pheatmap”: Pretty heatmaps. Version 1012. 2019;1–8. (18)*

*Kassambara A, Mundt F. factoextra: Extract and Visualize the Results of Multivariate Data Analyses [Internet]. 2020. Available from:* [*http://www.sthda.com/english/rpkgs/factoextra*](http://www.sthda.com/english/rpkgs/factoextra) *(20)*

**2)** Adding titles to the figures would help the reader to follow (e.g.: add adenocarcinoma above the corresponding correlogram, ...)

As the Reviewer asked, we completed our bioinformatic figures with titles to help the reader to follow the results **(Figure 6 panels C and D, S7 Fig panels A and B)**.

**3)** Improve the title of figure 6 "PCA" which does not take into account the half of the figure.

As the Reviewer asked, we completed our bioinformatic figures with altered titles to help the reader to follow the results **(Figure 6A and S6A Fig)**.

**4)** Figure2: results observe using MFI histograms and the barplots are sometimes different, perhaps more representative MFI histograms should be used instead (e.g.: HLA-ABC of WM983B cell line shows clear differences by MFI plot at top of Fig 2A but much more moderate typical effect in barplot below).

The representative histograms shown in the figures represent the results of 1 donor for each parameter. Unfortunately, we cannot show an “ideal” donor in which every change perfectly reflects the overall results shown in the bar graphs.

**5)** “Our result suggests that tumor cell lines and dexamethasone alter the epigenetic regulation of the CD14 and DC-SIGN expression in moDCs. All tumor cell type-derived factors induced the co-expression of CD14 and DC-SIGN.” (lines 495-498): Authors say cell lines and dexamethasone alter the epigenetic regulation of CD14 and DC-SIGN expression in moDCs, however, only CD14 expression is altered as described after, maybe this sentence could be reformulated to explain better this observation.

As the Reviewer recommended we reformulated the sentence; *“Our results suggest that tumor cell supernatants and dexamethasone alter the epigenetic regulation of moDCs CD14 expression in different ways.”*

*lines (305-306)*

**6)** “We have found that the expression pattern of MHCII and PD-L1 was also correlated with the ability of TU-CM-conditioned moDCs to stimulate CD4+CD25+FoxP3+ T cells. (line 545-547) : Authors could be precise these observation is in the melanoma cell lines.

Taking into account the newly obtained experimental results, the results were re-analyzed and the 'Results' section was rewritten (Lines 456-519). Because of the generation of new results, discussion was also altered in general, as the correlations were changed.

**7)** “Overall, TU-CM-educated DCs induced Treg differentiation, especially the subtype of IL-10 producing Tregs.” (lines 554-555): Add the figure reference.

Appropriate Figure references are already labelled in the text.

**8)** “… Interestingly, this correlation was the opposite in the case of melanoma cell-line-CM-conditioned moDCs” (lines 576-577): Authors precise the TNFα and CD4+CD25+FoxP3+ T cells positively correlate in melanoma cell lines, however, only WM1617 cell line expresses TNFα well. Authors should describe this observation more carefully.

Taking into account the newly obtained experimental results, the results were re-analyzed and the 'Result' section was rewritten (Lines 456-519). Because of the generation of new results, discussion was also altered in general, as the correlations were changed.

**9)** “IL-8 production by adenocarcinomas negatively correlated with the expression of MHC and costimulatory molecule CD86 as well as IL-10 producing helper T cell polarizing capacity of moDCs” (lines 585-587): It is only the case with MHCII, not MHCI.

Taking into account the newly obtained experimental results, the results were re-analyzed and the 'Result' section, was rewritten (Lines 456-519). Because of the generation of new results, discussion was also altered in general, as the correlations were changed.

**Suggestions:
1)** Unless I have not seen the file, it would be useful to provide an excel or csv file containing all the variables used for the bioinformatics analyses.

Thank you for your constructive suggestions! As the Reviewer asked, we provide a table containing the average values of the variables used for the bioinformatics analyses:

Z-score values were calculated from the raw data and a column-scaled heatmap **(Figure 6B and S6B Fig)** was generated.

**2)** For the correlation analyses, adding in additional figures the plots of correlations of the most interesting pairs of variables could help to better understand the data. Moreover, it could allow to see if some melanoma or adenocarcinoma cell lines behave differently from others.

The control samples were removed from the analysis shown in Figure 6, so that the differences and similarities between each tumor cell line were better revealed. A heatmap has also been added to the figure for easier interpretation **(Fig 6B)**. In this way, it became visible that some melanoma or adenocarcinoma cell lines behave differently from others. More precisely, WM1617 metastatic melanoma cell line has the same effect on the moDC’s function as the adenocarcinomas, but it is quite differ from the other melanomas. ‘Results’ has already corrected (Lines 456-519) and the observation is mentioned in the discussion also (Lines 602-604).

“*Additionally, WM1617 metastatic melanoma cell line has the same effect on the moDC’s function as the adenocarcinomas, but it is quite differ from the other melanomas.”*

**3)** To complete PCA, authors could be produce a a clustering/heatmap on the same data.

As the Reviewer asked, we completed the bioinformatic results with heatmaps for the easier and more spectacular interpretation of our results **(Figure 6B and S6B Fig)**.

**Reviewer #2**:

**Major comments:**

**1.** In figure 1, the authors define mo-DC as CD14+ or CD209+. However, CD14 and CD209 are not specific markers of mo-DC and can be expressed by macrophages too. Moreover, it has been shown by mass cytometry that the mo-DC population (differentiated from monocytes with GMCSF and IL-4) is homogeneous, with some cells expressing CD163 and MERTK suggesting the presence of mo-mac in this model (Sander, Immunity 2017). Are the authors sure that tumor cell derived factors modify only surface markers and not the identity of the cells? In line with this comment, the authors show that CD1a (typical mo-DC marker) expression is decreased by factors secreted by tumor cells.

We are grateful to the Reviewer for this comment or perception. We performed a further phenotypic analysis of monocyte-derived cells differentiated in the presence of TU-CMs. We examined the expression of CD163 and CD206 macrophage markers. We present the new results about the CD163 and CD206 expression profile of TU-CMs-altered monocyte-derived cells introducing a new supplementary figure **(S4 Fig).**

We rewrote the appropriate paragraphs of the ‘Result’ section as follows: (lines 314-328)

*“Considering the alternative hypothesis, indeed, the phenotype and properties of the monocyte-derived cells differentiated in the presence of conditioned media is consistent with the monocyte-derived macrophages. We performed further phenotypic analysis of monocyte-derived cells differentiated in the presence of TU-CMs (S4 Fig). We found, that WM983B significantly up-regulated the expression of CD163, in which condition the expression of DC-SIGN and CD1a was significantly silenced, however only the small percentage of the cells expressed the CD163 molecule. As a conclusion, WM983B-produced-CM promote the differentiation of CD1a-DC-SIGNmed monocyte-derived cells with a small population of CD163+ macrophage-like cells. However, the expression of CD206 was significantly diminished on the surface of WM983B-CM-conditioned monocyte-derived cells compared to the control moDCs (RPMI). Additionally, in line with the literature, differentiation of monocytes in the presence of IL-4, GM-CSF and dexamethasone induces the increased expression of CD163 (Octavio Morante-Palacios, Nucleic Acids Research, 2022).* *As a conclusion, WM983B-produced-CM promotes the differentiation of CD1a^-^DC-SIGN^med^ monocyte-derived cells with a small population of CD163^+^ macrophage-like cells*. “

New reference: (30)

“*Morante-Palacios O, Ciudad L, Micheroli R, De La Calle-Fabregat C, Li T, Barbisan G, Houtman M, Edalat SG, Frank-Bertoncelj M, Ospelt C, Ballestar E. Coordinated glucocorticoid receptor and MAFB action induces tolerogenesis and epigenome remodeling in dendritic cells. Nucleic Acids Res. 2022;50(1):108–26.”*

**2.** Figure 2 : it is not clear how the authors define the positive population for the different markers. In comparison with the unlabeled control, it seems that almost all the cells are positive. They should rather analyze the MFI.

Thank you for this constructive comments. According to the Reviewer’s suggestion, we have inserted a new supplementary figure into the revised manuscript **(S5 Fig)** examining the MFI values. Similar patterns can be seen in Figure 2 and the new Figure S5, but we consider the method used is suitable for comparison, especially for non-homogeneous cell populations.

We modified the text as follows: (lines 348-349)

*“The frequency of positive/highly positive cells are presented on Fig 2 and MFI values on S5 Fig.”*

**3.** Figure 3 : the authors show that some tumor cell lines secrete IL8 and IL6. When measuring the secretion of cytokines by mo-DC, did the authors use a control to exclude cytokines coming from the conditioned medium?

We apologize for the incorrect description of the figure. Figure 3 panel A depicts the soluble mediators secreted by monocyte-derived cells, and panel B shows the secretion profile of tumor cells. Panel B of Figure 3 shows the soluble mediator content of TU-CMs immediately prior to use for the monocyte culturing.

**4.** In figures 4 and 5, the authors should increase the n. It is hard to reconcile how they obtain statistically significant results with n=3.

According to the Reviewer’s suggestion, we repeated our experiments. In the revised version of the manuscript **Figure 4** shows the data from 7, and the **Figure 5** depicts the results of 4-6 donors. This information is updated in the Figure legend.

After these experiments the bioinformatical analyses were performed again, to take into account the newly generated data **(Figure 6, S6 and 7 Figs)**.

**5.** In figure 6, the staining for Foxp3 should be improved. A better staining allowing the identification of T regs could modify the conclusion.

We agree with the Reviewer that FoxP3 staining could be better, however as the staining with PerCP and APC parallel generates compensation problems. Therefore, and due to other technical difficulties, we cannot improve FoxP3 staining. These data were excluded in the revised version of the manuscript. This change does not affect our conclusion, as more marked differences were observed for the other markers. In line with this, we re-analyzed our data without the variable ‘CD4^+^CD25^+^FoxP3^+^ T cells’ for the bioinformatic-based figures **(Figure 6, S6 and 7 Figs)**.

**Minor comments:**

1. In the introdcution, the authors should mention the DC1 population. It has been shown that this subset of DC is involved in the anti-tumor responses.

As the Reviewer asked, in the revised version of the manuscript, the introduction has been completed as follows (lines 92-94):

“*Although, all major subsets of DCs are critical components of anti-tumor immunity, Irf8- and Batf3-dependent cDC1 (Xcr1+CD103+) specialize in antigen cross-presentation with MHC class I (MHCI) molecules for activating CD8+ T cells (Iwanowycz, JCI Insight., 2021)*.”

New reference: (8)

“*Iwanowycz S, Ngoi S, Li Y, Hill M, Koivisto C, Parrish M, Guo B, Li Z, Liu B. Type 2 dendritic cells mediate control of cytotoxic t cell resistant tumors. JCI Insight.”*

2. 2. In the section « DC-T cell co-cultures » of the material and methods, more details about the procedure are needed. How were T cells isolated? Were CD8 and CD4 T cells cultured together with mo-DC (3 cell types in the same well)? Did the authors use frozen PBMCs to isolate T cells (as they fo autologous co-cultures)?

According to the Reviewer’s recommendation, in the revised version of the manuscript we give a more detailed description of the T cell-related steps in ‘materials and methods’: (lines: 178-186)

*“PBMCs were separated from buffy coats by Ficoll-Paque Plus gradient centrifugation. According to the manufacturer's protocol, monocytes were purified from PBMCs by positive selection using immunomagnetic anti-CD14-conjugated microbeads. At the end of this process, the monocyte-depleted peripheral blood lymphocytes (PBL) were frozen immediately after the separation. The cells were used for the T cell polarization assay, as autologous PBL including helper and cytotoxic T cells as well.* *On the day 4 of monocyte differentiation, monocyte-derived cells were whashed and they were cocultured with* *freshly thawed autologous PBL (1×10^5^ DC :1×10^6^ PBL).”*

Thus, monocyte-derived cell – PBL cocultures basically contained CD4 and CD8 positive T cells as well.

3. The authors should specify which statistical tests were performed.

According to the Reviewer’s recommendation, in the revised version of the manuscript we provide a more detailed description of statistical analyzes as follows: (lines 269-273)

“*One-way ANOVA followed by Tukey’s post hoc test was used for comparisons for more than 2 groups. The results were expressed as mean +SD. Analyses were performed by using Excel (Microsoft Corporation) and GraphPad Prism Version 6.0 (GraphPad Software Inc.) software. Differences were considered to be statistically significant at P < 0.05. Significance was indicated as *P < 0.05; **P < 0.01; ***P < 0.005; ****P < 0.0001*.”

4. In figure 1 and 2, the authors should include an FMO for each condition and not only the control one (RPMI).

We agree with the reviewer's request to also show the autofluorescence of the cells conditioned in different ways. We present these data introducing a new supplementary figure **(S3 Fig).** We supplemented the text to indicate that conditioning monocytes with TU-CMs during the differentiation process had no effect on cell autofluorescence. However, we have not supplemented the histograms with the FMO curves in figure 1 and 2 to avoid data overcrowding, which may make it difficult to interpret our results.

The text was completed: (lines 286-287)

*“None of these treatments modified DC viability (S2 Fig) and the conditioning of monocytes with TU-CMs during the differentiation process had no effect on the autofluorescence of the cells (S3 Fig)*.”

5. The authors should explain why they choose to investigate the phagocytosis of bacteria in the context of anti-tumor immunity.

In our comparative analysis, we managed to identify different correlations between the cell surface markers and phagocytosis of L. casei by moDCs which observations corroborate the importance of the simultaneous consideration of variables. As an important microbiota component, lactic acid bacteria present in the gut have been shown to have a role in the regression of carcinogenesis due to their influence on immunomodulation. Smits et al. demonstrated that L. casei binds to DCs and can actively prime DCs leading to the activation of IL-10 production by regulatory T cells. Different tumor cells can manipulate the uptake of probiotics and thus regulate their potential therapeutic effect.

6. In figure 5, when gating T cells, the authors should not exclude the cells with higher FSC and SSC. After proliferation, T cells become bigger and more granular.

Thank you very much for drawing our attention to these mistakes! In the revised version of the manuscript, we corrected the gating strategy, and we re-analyzed our results taking into account the previously excluded cells. Newly generated bar graphs show the revised data as well as the new statistical analysis **(Figure 5)**. We also performed the new bioinformatic analysis taking into account the new data **(Figure 6, S6 and 7 Figs)**.

7. In figure 5D, it is not clear why the authors gate on two different populations (black and red).

As we re-organized the **Figure 5**, double gating has been removed. Cytokine production of CD4^+^CD25^+^ cells is visualized on histogram with gating the IL-10^+^ cells.

8. I would recommend the authors to improve the language to facilitate the readability of the text.

As requested by the reviewer, we have tried to improve the language of the manuscript.
