# Supplementary material for: Comprehensive analysis of different tumor cell-line produced soluble mediators on the differentiation and functional properties of monocyte-derived dendritic cells
Source: PLoS One. 2022 Oct 4;17(10):e0274056. doi: 10.1371/journal.pone.0274056 (PMC9531813; doi:10.1371/journal.pone.0274056)
Supplement: Attachment — Submitted filename: Response to Reviewers.docx [file pone.0274056.s009.docx]

**Response to Reviewers**

Thank you very much for the review of our manuscript entitled *“Comprehensive analysis of different tumor cell-line produced soluble mediators on the differentiation and functional properties of monocyte-derived dendritic cells”* and the opportunity to resubmit a revised version.

We greatly appreciate the positive evaluation of our manuscript and the valuable comments of the Reviewers. We addressed all the comments of the reviewers. To enhance the quality of our manuscript we have conducted additional experiments and made several changes in the text. Please see our detailed responses below.

**Reviewer#1**:

1) References should be added for statements on lines 100-107 and lines 627-628.

First of all, we are very grateful to the reviewer for a thorough review of our manuscript. Additionally, we thank you for bringing errors to our attention. We apologize for the inconsistencies between the text and the results presented in the figures. We corrected the highlighted sentences, refined the statements and conclusions. We checked the consistency of the text and figures as well as the text were edited in the revised manuscript. In the revised version of the manuscript, the text was aligned with the figures and panels in all cases.

We completed the text with the next references:

line 108:

*Krempski J, Karyampudi L, Behrens MD, Erskine CL, Hartmann L, Dong H, Goode EL, Kalli KR, Knutson KL. Tumor-infiltrating programmed death receptor-1+ dendritic cells mediate immune suppression in ovarian cancer. J. Immunol. 2011;186:6905–6913.*

line 635:

*Itakura E, Huang RR, Wen DR, Paul E, Wunsch PH, Cochran AJ: IL-10 expression by primary tumor cells correlates with melanoma progression from radial to vertical growth phase and development of metastatic competence. Mod Pathol. 2011, 24: 801-809. 10.1038/modpathol.2011.5.*

2) The description of the methods concerning the bioinformatics part is really much better. However, it is still necessary to add the parameter values used for these commands, even if just the default values were used. Similarly, the parameter values for pheatmap in figure 6 and S6 should be specified.

As requested by the reviewer, we have completed the M&M with the specified parameter values: lines 256-272

Bioinformatical Correlation Analyses

R (version 4.1.3) [16] and RStudio (version 1.4.1717) were used for bioinformatical analyses. Dataset contained the mean of marker expression, cytokine secretion, T-cell polarizing and phagocytosis data. Correlation matrices were performed by *R base* package (v 4.1.3) with *cor* function for adenocarcinoma and melanoma cell lines-conditioned moDCs separately. Correlograms from correlation matrices were plotted by *corrplot* (v 0.92) package [17] with *corrplot* function. P-value matrix and confidence intervals matrix were added using the *cor.mtest* function of *corrplot* package. Significance levels were defined as *P < 0.05; **P < 0.01; ***P < 0.001. Mixed correlograms were performed by *corrplot.mixed* function of the *corrplot* package. Heatmap was generated from the raw data by *pheatmap* function of *pheatmap* (v 1.0.12) library [18]. Principal Component Analysis (PCA) was made by *PCA* function of *FactoMiner* package (v 2.4) [19] from raw data with or without controls (RPMI and dexDC) separately. Results of the PCA were visualized by *factoextra* package (v 1.0.7) [20] with *fviz_pca_ind* function.

is completed as it is highlighted below:

**Bioinformatical Analyses**

R (version 4.1.3) [16] and RStudio (version 1.4.1717) were used for bioinformatical analyses. Dataset contained the mean of marker expression, cytokine secretion, T-cell polarizing and phagocytosis data. Correlation matrices were performed by *R base* package (v 4.1.3) with *cor* function for adenocarcinoma and melanoma cell lines-conditioned moDCs separately. Correlograms from correlation matrices were plotted by *corrplot* (v 0.92) package [17] with *corrplot* function using the following parameters: type = upper, order = original. P-value matrix and confidence intervals matrix were added using the *cor.mtest* function with confidence level 0.95 of *corrplot* package. Significance levels were defined as *P < 0.05; **P < 0.01; ***P < 0.001. Mixed correlograms were performed by *corrplot.mixed* function of the *corrplot* package with the parameters lower = number, upper = circle, order = original. Heatmap was generated from the raw data by *pheatmap* function of *pheatmap* (v 1.0.12) library [18] using the scale = column parameter. Principal Component Analysis (PCA) was made by *PCA* function of *FactoMiner* package (v 2.4) [19] using the default values of scale.unit, ncp and graph parameters from raw data with or without controls (RPMI and dexDC) separately. Results of the PCA were visualized by *factoextra* package (v 1.0.7) [20] with *fviz_pca_ind* function with default parameters.

3) The text describing the results in Figure 3 is unclear.

We apologize for the inconsistencies between the text and the results presented in the figures.

The statement quoted in line 371-372 should reference Figure 3B instead of 3A. Conversely, the statement on line 381-382 should refer to Figure 3A.

As it is labelled in the revised manuscript, we corrected the highlighted sentence.

Similarly, the statement in line 378-381 concerning WM1617 should refer to Figure 3A instead of 3B.

As it is labelled in the revised manuscript, we corrected the highlighted sentence.

4) Figure 4B legend should be revised to refer to boxplots instead of histograms.

Mentioned sentence is corrected in the revised manuscript:

“*Diagram shows the mean plus SD of seven independent experiments. Boxplot shows one of the seven independent experiments*.”

5) Figures 5 and 6 appear to have been swapped or mislabeled. Eg lines 422- 451 appear to refer to the figure labeled 6 instead of 5 and lines 453-530 refer to the figure labeled 5 instead of 6.

In this case we did not find mistakes, however, in the automatically generated pdf file the sequence of Figure 5 and 6 was different, but the numbers of the given figures were correct. In this regard, we will carefully check the merged uploaded files.

6) The two sentences on line 460-463 should be joined and reworded for clarity.

We apologize for the incorrect wording. Mentioned sentence is corrected in the revised manuscript:

“*To reveale differences in the strategy of melanomas and adenocarcinoma cell lines in the manipulation of moDC differentiation*.”

is changed to:

“*To reveal differences in the strategy of melanomas and adenocarcinoma cell lines in the manipulation of moDC differentiation bioinformatic analysis was performed*.”

7) Several errors are present in the description of the correlation analyses of adenocarcinomas: CD1a and HLA-ABC are not positively correlated (line 475), CD1a is not negatively correlated with IL10-producing CD4+CD25+ but is negatively correlated with IFNγ-producing CD8+ cells (line 489) and HLA-DR is not negatively correlated with IFNγ-producing CD8+ but with IL8-production (line 493-494). In addition, t might be better to specify the correlations grouped in the term “T cell stimulatory potential” (line 513), such as: “…T cell stimulatory potential (IFNγ-producing and T cell activator)”.

We apologize for the inconsistencies between the text and the results presented in the figures.

In the corrected manuscript the next changes are labelled:

“*CD1a and CD1d, HLA-ABC, PD-L1, phagocytic activity, and TNFα/ IL-6/ IL-8-production*”

is changed to:

“*CD1a and CD1d, PD-L1, phagocytic activity, and TNFα/ IL-6/ IL-8-production*”

Additionally:

“*the CD1a and HLA-ABC, HLA-DR, CD86, IL-10 secretion and IL-10-producing CD4+CD25+ cells activatory capacity*”

is changed to:

“*the CD1a and HLA-ABC, HLA-DR, CD86, IL-10 secretion and IFNγ-producing CD8+ T cells activatory capacity*”

Additionally:

“*the HLA-DR and phagocytic activity, TNFα-production and IFNγ-producing CD8+ T cells activatory capacity*”

is changed to:

“*the HLA-DR and phagocytic activity, TNFα-production and IL-8 production*”

Additionally:

*“CD209, CD1a, HLA-ABC, HLA-DR, PD-L1, IL-10 or TNFα and IL-8 production and T cell stimulatory potential*”

is changed to:

“*CD209, HLA-ABC, HLA-DR and IL-8 production and IFNγ- or IL-10 producing T cell stimulatory potential*”

8) There are some small errors in the discussion:

There is an error in line 622, WM278 should be replaced by WM1617.

The mistake is corrected in the revised manuscript.

*“In our system, only DCs treated with the conditioned media of WM278 and WM983B metastatic melanoma cell lines decreased the secretion of IFNγ by CD8+ T cells compared to its primary counterpart.”*

is changed to:

*“In our system, only DCs treated with the conditioned media of WM1617 and WM983B metastatic melanoma cell lines decreased the secretion of IFNγ by CD8+ T cells compared to its primary counterpart.”*

There is an error in the sentence of line 638-639: the negative correlation between TNFα secretion and IL-10 producing CD4+CD25+ T cells is observed in melanoma and not in adenocarcinoma.

The mistake is corrected in the revised manuscript.

“*In oppose to the moDCs conditioned by melanomas, in the group of adenocarcinoma-CM-educated moDCs TNFα secretion negatively correlated with the ability of moDCs to induce IL-10 producing CD4+CD25+ T cells*.”

is changed to:

“*In the group of melanoma-CM-educated moDCs TNFα secretion negatively correlated with the ability of moDCs to induce IL-10 producing CD4+CD25+ T cells*.”

9) Several typos are present in the manuscript such as: "DC-SIGN/CD20916" (line 553) instead of "DC-SIGN/CD209", "DCsand" (line 589) instead of "DCs and", etc. Extensive proof reading is required.

As requested by the reviewer, we have corrected the mentioned mistakes and we read the entire text. We checked the consistency of the text and figures as well as the text were edited in the revised manuscript.

**Reviewer #2:**

The flow cytometry data still needs to be improved.

In figure 2 : it is still not clear how the authors define the positive population for the different phenopytpic markers. If the positive population is determined according to the unlabelled cells, the mean should be higher than 80% (and not 50% as shown in the graphs).

Unlabeled cells are represented by empty histograms in Figure 2, the mean of the positive population exceeds 80% of this in all cases. The gray histograms representing RPMI-DCs provided the basis for comparison. However, for the investigated markers, in most cases, a population expressing the given molecule at a higher level (high) appeared, the percentage of which was presented in the bar charts. Comparison with only unstained or control samples or plotting the MFI would have masked the presence of cells highly expressing the markers. For complex and more objective interpretation of data, MFI values are presented on S5 Fig.

In figure s4 : a representative facs plot should be shown for CD163 and CD206.

As the Reviewer recommended, S4_Fig was completed with representative FACS results showing the expression levels of CD206 and CD163 markers. This Figure is re-uploaded to the website.

The new figure information is labelled in the text (Figure legend of S4_Fig):

*“Histograms show one of the three independent experiments.”*

In figure 4 : a negative control (monocyte-derived cells + Bacteria at 4°C) is lacking. The fluorescence measured should correspond to binding of the cell surface instead of phagocytosis.

We are grateful to the Reviewer for this comment or perception. As the Reviewer asked, we performed a further experimental analysis. These results are shown on Fig4 which is already uploaded to the website.

The Figure legend is completed with the description of the new measurement:

*“In vitro differentiated four-day monocyte-derived cells were coincubated with mCherry-labeled L. casei for 4h at a ratio of 1:10 at 37 °C and at 4 °C as a control. Phagocytic activity of moDCs was measured by detecting mCherry fluorescent protein-positive moDCs with flow cytometry after 4h.* *Diagram shows the mean plus SD of seven (37 °C) or three (4 °C) independent experiments.* *Boxplot shows one representative sample from the seven independent experiments.”*
